# Non-contrast fresh-blood MRA for assessment of abdominal endovascular stent grafts

**DOI:** 10.1186/1532-429X-11-S1-P229

**Published:** 2009-01-28

**Authors:** Timothy SE Albert, Connie Luna, Patrik Zetterlund, Mitsue Miyazaki

**Affiliations:** 1 Salinas Valley Memorial Hospital, Cardiovascular Diagnostic Center, Monterey, CA USA; 2 Salinas Valley Memorial Hospital, Salina, CA USA; 3 Central Coast Cardiology Research, Salinas, CA USA; 4 Toshiba Medical Research Institute, Vernon Hills, IL USA

**Keywords:** Abdominal Aortic Aneurysm, Abdominal Aortic Aneurysm, Abdominal Aortic Aneurysm Repair, Black Blood, Bright Blood

## Objective

Serial imaging of abdominal aortic endovascular stent grafts (AA-ESG) is done in order to determine: 1) stent stability, 2) presence of endograft leak, and 3) residual abdominal aortic aneurysm (AAA) sac size. CT angiography (CTA) has traditionally been used for serial follow-up. Given the high incidence of renal dysfunction in patients with AAA and potential contraindications to both CT and MR contrast agents we development a non-contrast vascular imaging protocol for assessment of patients with AA-ESG.

## Methods

**Figure 1:**
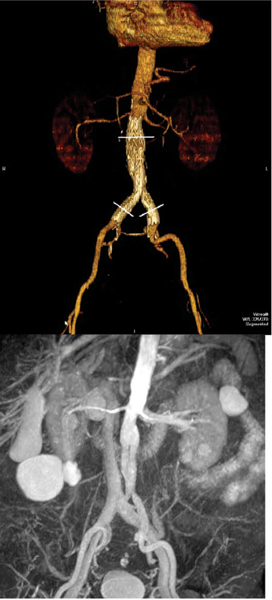
**CTA** ***(upper image*** **) and FBI** ***(lower image)*** **of same patient after AA-ESG**. The diastolic-triggered FBI image clearly depicts the lumen of the endovascular stent graft and its iliac sleeves in bright blood. The abdominal veins and large renal cysts are also well visualized.

Consecutive patients who had AAA repair with AA-ESG and had prior CTA for comparison were imaged with a combined black blood (BB) imaging protocol using 2D single-shot FSE for depicting vessel wall dimensions (aneurysm sac size) and a fresh-blood imaging (FBI) technique using 3D single-shot FSE for bright blood imaging of the vessel lumen (stent stability). Comparisons were made in accuracy of endovascular luminal assessment (FBI vs. CTA along 3 points, see Figure 1) and aneurysm wall-to-wall dimensions (BB vs. CTA). The non-contrast technique did not allow direct assessment of endograft leak. CTA was performed on a Toshiba Aquilion 64-slice CT scanner while MRI imaging was performed using a Toshiba Vantage Atlas 1.5 T MRI scanner.

## Results

Consecutive patients are reported on (n = 5): 73 ± 4 yo, 45 ± 16 months since AAA repair (mean ± SD). Aneurysm dimension at its greatest size as assessed by CTA (3.9 ± 0.1 cm) was similar to that for BB imaging (3.8 ± 0.1 cm) (p = 0.40). Endograft visualization as assessed by luminal dimensions was similar between FBI and CTA (r2 = 0.96, n = 15) with good visualization of the AA-ESG and surrounding arterial tree.

## Conclusion

A combined approach of 2D BB and 3D FBI imaging of AA-ESG can accurately assess aneurysm sac size and endograft angiographic characteristics. Although this non-contrast technique cannot directly assess for endograft leak this approach may still be an acceptable alternative to contrast imaging for serial follow-up of patients with renal dysfunction. Further validation of this novel technique will need to be done in a larger cohort of patients.

